# Identification and development of an independent immune-related genes prognostic model for breast cancer

**DOI:** 10.1186/s12885-021-08041-x

**Published:** 2021-03-30

**Authors:** Lin Chen, Yuxiang Dong, Yitong Pan, Yuhan Zhang, Ping Liu, Junyi Wang, Chen Chen, Jianing Lu, Yun Yu, Rong Deng

**Affiliations:** 1 Department of General Surgery, Jiangsu Cancer Hospital & Jiangsu Institute of Cancer Research & The Affiliated Cancer Hospital of Nanjing Medical University, Nanjing, 210009 China; 2 First Clinical Medical College of Nanjing Medical University, Nanjing, 210029 China; 3 Nanjing Medical University, Nanjing, 211116 China; 4 University of Chinese Academy of Sciences, Beijing, 100101 China; 5 Nanjing University of Chinese Medicine, Nanjing, 210029 China; 6 Department of Medical Informatics, School of Biomedical Engineering and Informatics, Nanjing Medical University, Nanjing, 211116 China

**Keywords:** Breast cancer, Immune genes, Prognosis, Risk scores model, Nomogram

## Abstract

**Background:**

Breast cancer is one of the main malignant tumors that threaten the lives of women, which has received more and more clinical attention worldwide. There are increasing evidences showing that the immune micro-environment of breast cancer (BC) seriously affects the clinical outcome. This study aims to explore the role of tumor immune genes in the prognosis of BC patients and construct an immune-related genes prognostic index.

**Methods:**

The list of 2498 immune genes was obtained from ImmPort database. In addition, gene expression data and clinical characteristics data of BC patients were also obtained from the TCGA database. The prognostic correlation of the differential genes was analyzed through Survival package. Cox regression analysis was performed to analyze the prognostic effect of immune genes. According to the regression coefficients of prognostic immune genes in regression analysis, an immune risk scores model was established. Gene set enrichment analysis (GSEA) was performed to probe the biological correlation of immune gene scores. *P* < 0.05 was considered to be statistically significant.

**Results:**

In total, 556 immune genes were differentially expressed between normal tissues and BC tissues (*p* < 0. 05). According to the univariate cox regression analysis, a total of 66 immune genes were statistically significant for survival risk, of which 30 were associated with overall survival (*P* < 0.05). Finally, a 15 immune genes risk scores model was established. All patients were divided into high- and low-groups. KM survival analysis revealed that high immune risk scores represented worse survival (*p* < 0.001). ROC curve indicated that the immune genes risk scores model had a good reliability in predicting prognosis (5-year OS, AUC = 0.752). The established risk model showed splendid AUC value in the validation dataset (3-year over survival (OS) AUC = 0.685, 5-year OS AUC = 0.717, *P* = 0.00048). Moreover, the immune risk signature was proved to be an independent prognostic factor for BC patients. Finally, it was found that 15 immune genes and risk scores had significant clinical correlations, and were involved in a variety of carcinogenic pathways.

**Conclusion:**

In conclusion, our study provides a new perspective for the expression of immune genes in BC. The constructed model has potential value for the prognostic prediction of BC patients and may provide some references for the clinical precision immunotherapy of patients.

**Supplementary Information:**

The online version contains supplementary material available at 10.1186/s12885-021-08041-x.

## Background

Breast cancer is one of the main malignant tumors that threaten the lives of women, which has received more and more clinical attention worldwide. It is regarded as the second common malignant tumors in the world. It occupied 25% of all malignant tumors [1]. Triple-negative breast cancer (TNBC) was supposed to be the foremost malignant sub-type, accounting for approximately 20% [2, 3]. It is manifested as a large tumor, a high level of differentiation, a high risk of metastasis, and lymph node invasion [4–6]. TNBC is characterized by negative human epidermal growth factor receptor 2 (HER-2), progesterone receptor (PR) and estrogen receptor (ER), thus resistant to endocrine therapy and trastuzumab [7]. Due to the lack of targeted treatment strategies, chemotherapy remained the unique treatment option [8]. Therefore, it is significant and urgent to conduct a comprehensive bioinformatics study on gene expression of breast cancer to identify potential genes that can be used as therapeutic targets in BC.

Previous evidence has shown that the immune system has a contradictory influence on the occurrence and development of cancer, contributing to both cancer progression and inhibition [9–12]. The immune system has a practical impact on the progress of BC. What’s more, the response of BC patients to immunotherapy and traditional treatment is interfered by immune system [13, 14]. However, immune evasion remains a tough problem in the immunotherapy of BC, which brings a big challenge for the treatment for BC and improvement of prognosis of patients. Since there are significant diversities in the expression profiles of immune genes between BC and other cancer types [15, 16], further research is needed to determine which immune genes can play a role as therapeutic targets.

In this study, we explored the lineage and expression profiles of immune genes in BC and its impacts on the prognosis of BC patients. Besides, the functional features as well as mutated features of these immune-related genes were described. Furthermore, 15 immune-related genes closely associated with overall survival were selected and an independent risk score model was constructed for the prognosis of BC. Moreover, a nomogram was also constructed to further explore the model’s ability to predict.

## Methods

### Acquisition of data

First of all, a list of 2498 immune genes were downloaded from ImmPort database. Additionally, the gene expression profiles of BC patients was obtained from the TCGA database (https://portal.gdc.cancer.gov/), including 112 normal cases and 857 tumor cases. Meanwhile, corresponding clinical data were also obtained (Table 1). |LogFC | > 1 and *P* < 0.05 were used as the criteria for screening differential genes. Because TCGA is an open and publicly available database, ethical approval is not required.

**Table 1:** Baseline clinical characteristics of samples

Variables	Total (*n* = 857)	Training cohort (*n* = 577)	Validation cohort (*n* = 280)
Age (year)			
< 60	484	325	159
≥60	373	252	121
Sex			
Female	846	570	276
Male	11	7	4
Stage			
I	151	99	52
II	501	336	165
III	188	134	54
IV	17	8	9
T stage			
T1	227	162	65
T2	510	332	178
T3	92	64	28
T4	28	19	9
N stage			
N0	411	285	126
N1	292	186	106
N2	102	73	29
N3	52	33	19
M stage			
M0	840	569	271
M1	17	8	9
Survival			
Dead	118	83	35
Alive	739	494	245

### Gene function enrichment analysis

Gene Ontology(GO) enrichment analysis is conducted to comprehend the biological process and molecular function of the differential genes, while Kyoto Encyclopedia of Genes and Genomes(KEGG) enrichment analysis is applied to identify potential related biological pathways. Gene enrichment analysis (GSEA) is performed between normal tissues and BC tissues in order to probe the biological pathways associated with immune genes risk scores.

### Construction and validation of the immune genes risk scores

Cox regression tool was used for survival analysis. On the basis of differential expression, single factor cox significant and survival-related prognostic immune genes were screened out. Further, the least absolute shrinkage and selection operator (LASSO) regression analysis is execute to reduce the dimensionality, so as to screen out the optimal variables. Based on the variables obtained by LASSO and the corresponding regression coefficients, the risk scores were calculated. The median value was utilized to divide patients into a high- and a low-risk scores group. The prognostic correlation of immune gene risk score was obtained by Kaplan-Meier curve. The credibility and predictive value of the risk scoring model was evaluated through time-related ROC curve.

### Analysis of copy number variation data and gene mutation analysis

Based on TCGA breast cancer data, the copy number variation (CNV) was analyzed using R-Circos package and R-ggplot2 package. Furthermore, the online tool website-cbioportal was used to analyze the genetic variation of hub genes. The threshold used was *P* < 0.05.

### Statistical analysis

R3.6.1 was used for statistical analysis. The independent t test was used for continuous variables with normal distribution, and the Mann-Whitney U test was used for continuous variables with skewed distribution. A two-sided test was used, and a *P* value of < 0.05 was considered statistically significant.

## Results

### Differentially expressed immune genes (DEIGs) screening of BC

**Fig. 1:**
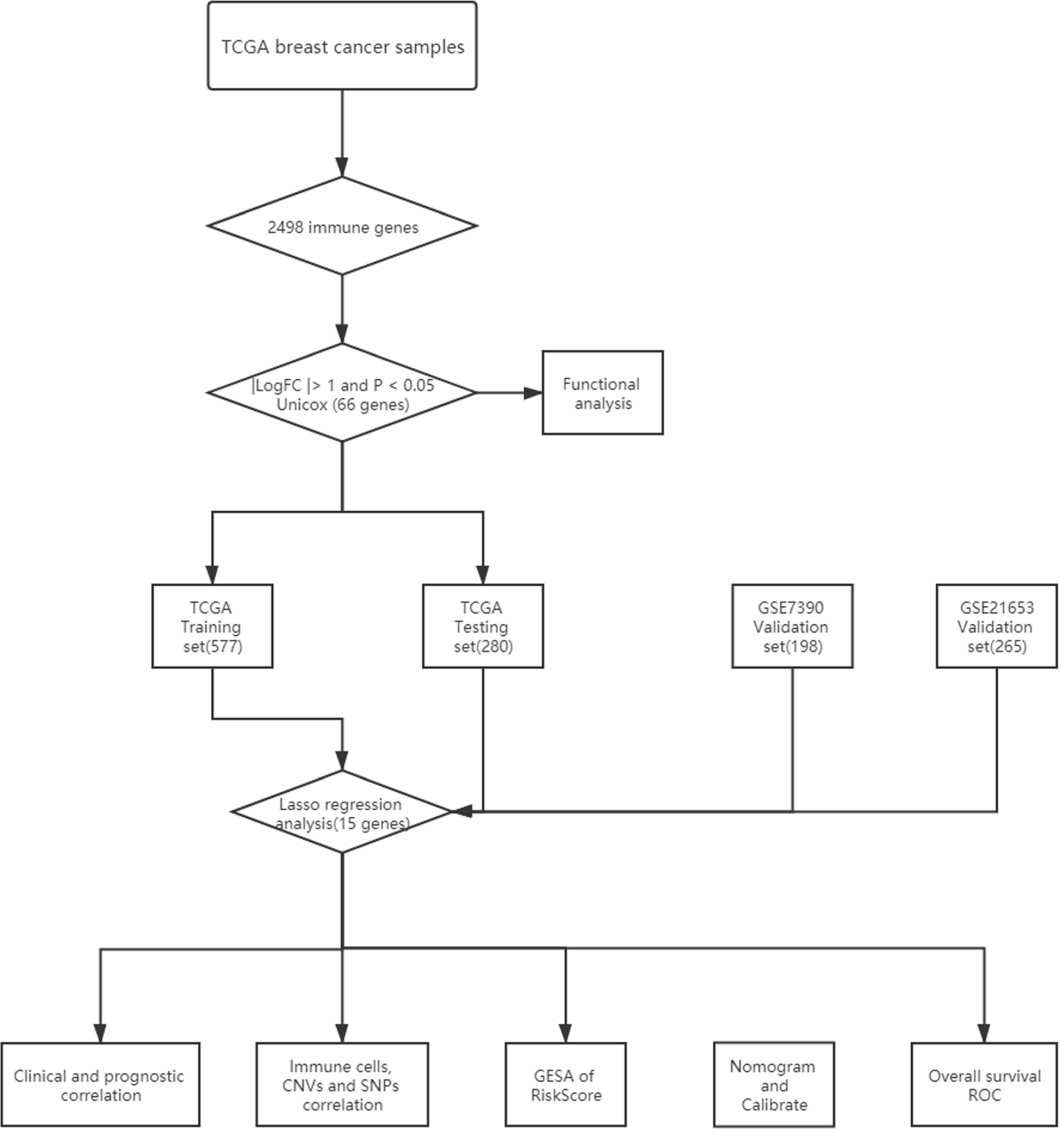
Flow chart of research design

**Fig. 2:**
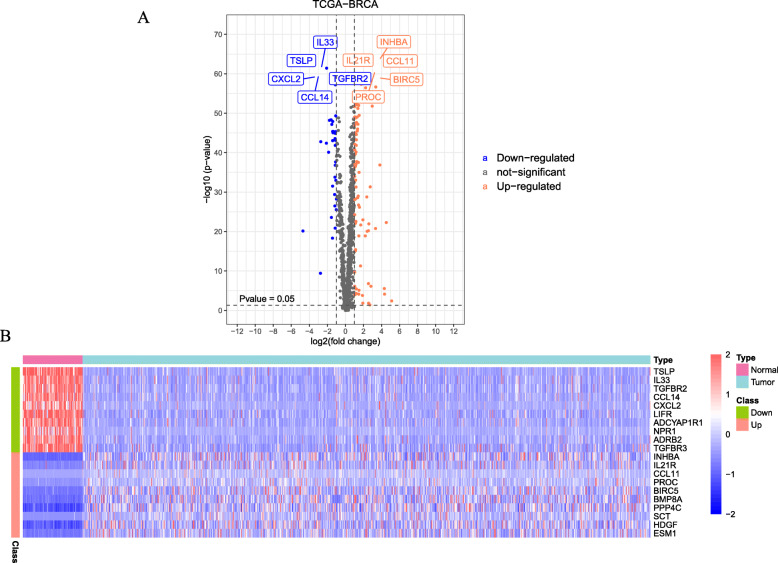
Identification of DEIGs. **a** volcano plots of 556 DEIGs in breast cancer and normal tissues from TCGA database. **b** Heatmap plots of top 10 up-regulated and top 10 down-regulated DEIGs. The colors in the heatmaps from green to red represent expression level from low to high. The red dots in the volcano plots represent up-regulation, the green dots represent down-regulation and black dots represent genes without differential expression

We designed a protocol for the analysis and construction of the prognostic model (Fig. 1). The analysis process was carried out in strict accordance with the protocol. A list of 2498 immune genes was obtained from ImmPort database. What’s more, the mRNA expression data of 857 cases of breast cancer and 122 cases of normal tissues were also obtained from the TCGA database for further bioinformatics analysis. The clinical features of samples were provided in Table 1. A total of 556 immune genes were determined as DEIGs between BC tissues and normal tissues, including 402 up-regulated and 154 down regulated (*p* < 0.05, Fig. 2a, Table 2). The heatmap spread out the top 10 up-regulated and top 10 down-regulated DEIGs (Fig. 2b).

**Table 2:** Analysis of differentially expressed immune genes in TCGA breast cancer

Up regulated	RAC2, ITK, ADAR, RFXANK, GDF11, IRF1, CMTM1, UCN2, MX2, TGFB1, KRAS, IFNA17, PLXNC1, IFNA2, DHX58, ADRM1, PIK3R3, PRDX1, SOCS1, S100A11, HLA-DQA1, VEGFA, F2R, OGFR, PSMC4, SEMA4A, IL1RN, HLA-DQB1, IL32, CALR, CD48, INHBC, DEFB104B, TNFRSF12A, CD3E, CCR6, SERPINA3, HSPA2, IL31RA, NR1I3, NFKBIB, INHBE, ISG20L2, IFIH1, LEAP2, CACYBP, ZAP70, CXCR4, GIPR, TNFRSF13C, TGFB3, DEFB103A, DDX58, GNRH2, CCL25, RFX5, OPRL1, SRC, NCR2, LAT, PSMD14, S100A16, IGF1R, NCR3, BCL3, HLA-DQA2, CARD11, RELB, CD79A, MCHR1, CD86, RBP5, IFITM1, UNC93B1, IL2RA, PTPN6, RLN1, FASLG, STAT1, PGC, MAPT, PSME2, AQP9, IRF5, IL2RG, HDGF, CCL19, BMP10, NFATC4, LYZ, RBP1, DEFB105A, PTGER1, LCK, TFRC, SH2D1A, CD3D, IL12RB1, RARRES3, IFNA5, MSR1, KIR3DL2, AMBN, PDF, HNF4G, KLRC2, SEMA3F, IFNA14, CCR5, CD1E, HAMP, IL23A, FCGR3A, BST2, CD22, SPAG11B, TMSB15B, GREM1, VAV2, PPP4C, ITGAL, RARA, NR2F6, PAK1, CXCL13, HLA-G, PRLR, TNFSF13B, PLAU, CD72, BLNK, MDK, PAK4, PSMD3, SLC29A3, FGF22, SEMA5B, IL2RB, NFKBIE, APOBEC3H, CSHL1, MC1R, SLC10A2, PAEP, MC4R, RLN3, CSF3R, IRF9, BMP15, GNRHR, ISG20, IFNA21, DEFB136, DEFA5, PLXNA3, INSL5, VAV3, CCR3, GUCA2A, PNOC, TOR2A, TAP1, BMP8B, RAC3, CLEC11A, CSPG5, IL18, IKBKE, RABEP2, IFNA13, CTLA4, TNFRSF4, NOX3, FAM19A5, IFI30, MIF, CBLC, NOX5, GPR33, RNASE2, EPGN, GPHB5, IFNW1, OAS1, CD1B, FLT3, INS-IGF2, NOX1, TG, NR2E3, MICB, SECTM1, GZMB, SEMA7A, CD19, IL24, C8G, MBL2, HSPA6, OSM, AGT, MX1, IL17F, HNF4A, SDC1, RSAD2, APOBEC3A, TYMP, HTR3E, ESR1vRETNvSLC11A1, PPY, CCL15, NOD2, DEFB121, UCN, PIK3R2, ANGPTL6, IFNA6, CGB2, AGRP, CCR7, CXCR3, PLAUR, CRABP2, IFNA7, DEFB129, RASGRP1, GDF2, IL3, LECT2, IFNG, FGFR4, LEFTY1, S100A7, ULBP2, WFIKKN1, RXFP1, MCHR2, IRF7, CCR4, COLEC10, AVPR1B, AZU1, PDCD1, TMSB15A, THPO, GALP, IDO1, CCL17, LTA, GALR3, MLN, IL11, TNFSF4, HTR3B, FSHB, RLN2, OBP2A, PRKCG, KIR2DL4, ICOS, SPP1, CGB8, IL1F10, INSL4, LTB, CELA1, FABP12, DEFB134, IL27, GALR2, SSTR2, PGLYRP2, RBP2, CXCR5, IFNA16, S100A14, ADM2, UTS2, IL12B, LCN12, OLR1, MMP9, CCL1, SCG2, IFNA4, MMP12, OASL, DEFB108B, IL9, AMELX, GDF15, IL9R, KCNH2, CTSE, DEFB110, FGFR3, CSH1, CCL20, MC2R, GPHA2, EDN2, TMPRSS6, GAL, SEMG1, BMP8A, PTH, ROBO2, RETNLB, HTR1A, DEFB128, PMCH, RXFP3, HRG, GH2, DEFB113, PTH2, IL21R, TNFRSF9, PROC, HTR3A, AMH, TNFRSF18, ESM1, MTNR1B, CXCL9, PYY, GCGR, INHA, CGB5, LCN9, DEFB112, ISG15, OPRD1, SLURP1, IFNA10, GDF9, CD1A, UMODL1, FGF23, ULBP1, IL17C, KIR3DL3, IL21, CXCL10, ARTN, INHBA, CCR8, BIRC5, SCT, VGF, TFR2, HTN3, SSTR5, IL20, PRLH, FGF21, GIP, R3HDML, CXCL11, KNG1, TUBB3, CCL7, S100A7A, LCN1, ORM2, APOH, EPO, PGLYRP4, FGF3, FGF5, IFNB1, PGLYRP3, BMPR1B, CCL11, FABP6, SEMG2, CAMP, S100P, MUC5AC, DEFB126, GHSR, DEFB123, DEFB115, ORM1, GCG, DEFB116, TRH, CSH2, FGF4, TCHHL1, IL19, HTN1, REG1A, PCSK1, IAPP, INS, CST4, CGA, UCN3
Down regulated	LEP, ADIPOQ, ACVR1C, FABP4, RBP4, DEFB132, OXTR, ANGPTL7, SAA1, ANGPTL5, CSF3, LALBA, CXCL2, BMP3, MASP1, NPR1, GLP2R, PENK, NOS1, PPARG, GDF10, ANGPT4, CCL14, TSLP, PLXNA4, SAA2, GHR, DES, ANGPTL1, CMA1, S100B, LHCGR, IL6, IL33, LEPR, FOS, SEMA3G, SCTR, FABP9, CX3CL1, PTN, CCL28, FGF2, ADCYAP1R1, STAB2, ADRB2, ANGPT1, EDN3, RXRG, CD209, LIFR, TGFBR3, RNASE7, CNTFR, AVPR2, OSTN, CCL21, TACR1, GNAI1, PF4, OGN, IGF1, PAK3, NTF4, GFAP, TGFBR2, IFNA8, NRG2, RBP7, APOD, CCL24, LCN6, KL, PTH1R, FGF1, BMP2, NGFR, EDNRB, GPR17, PTGFR, NR4A3, ELANE, S1PR1, CCL13, CCL16, CAT, CXCL12, IL17B, ANGPTL4, SOCS3, ACO1, NRG1, NR4A1, CYR61, LTBP4, NR3C2, PDGFD, CCL23, PPBP, SEMA3D, NPR3, NMB, SCGB3A1, ANGPTL2, TINAGL1, ESR2, CRIM1, CXCL3, NR3C1, MET, TEK, IL17D, BMP6, EGFR, VIP, CTSG, VIM, LRP1, GREM2, FGF7, PTGS2, JUN, PIK3R1, ROBO3, LCN10, IL17RD, TSHB, CSRP1, AHNAK, SEMA5A, PLA2G2A, MARCO, ADM, PMP2, FAM3D, TNFRSF10D, SEMA3A, SEMA6D, EDN1, NOV, PLTP, LGR6, PDGFRA, TLR4, SSTR1, AVPR1A, PDGFA, TPM2, PTGER4, THRB, EGF, IL11RA, CRHR2, CER1, ICAM2, A2M, PTGDS, TAC1, SLIT2, LGR4, BACH2, PDGFRL, C3, FGF16

### Functional annotation of these 556DEIGs

To study the potential mechanisms and molecular functions of the identified 556 DEIGs, we conducted the GO and KEGG analysis. The top three enriched GO terms for up-regulated DEIGs and down-regulated DEIGs were: T cell activation, lymphocyte differentiation and response to virus; cell chemotaxis, positive regulation of response to external stimulus and leukocyte migration, respectively (Fig. S1A). KEGG analysis revealed the top three enriched pathway for up-regulated DEIGs and down-regulated DEIGs were: Cytokine−cytokine receptor interaction, JAK − STAT signaling pathway and Chemokine signaling pathway; Cytokine−cytokine receptor interaction, JAK − STAT signaling pathway and EGFR tyrosine kinase inhibitor resistance pathway, respectively (Fig. S1B).

### Establishment of immune prognosis model

**Fig. 3:**
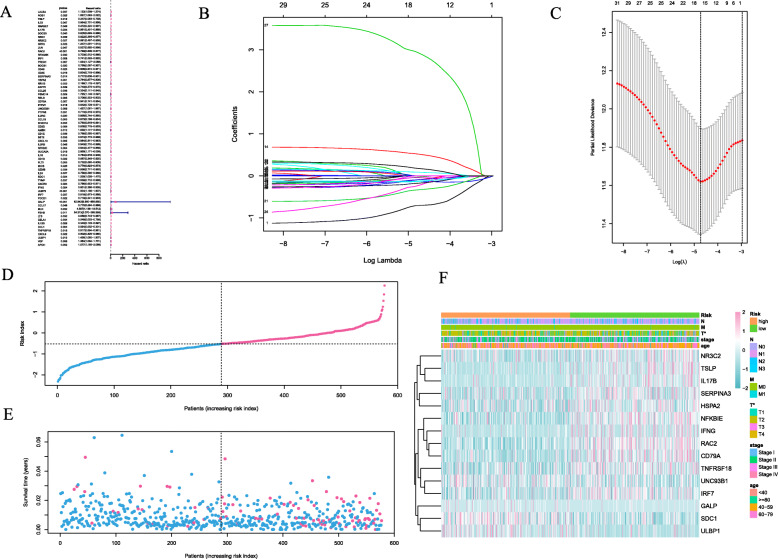
**a** Univariate survival analysis by Cox proportional hazards models to select prognostic key immune genes. **b**-**c** LASSO Cox regression model for19 prognostic immune genes used to construct immune genes risk score model. **d** Distribution of immune risk scores in breast cancer patients. **e** Distribution of survival status in breast cancer patients. **f** Distribution of specific risk factors in the high- and low-risk groups (divided by median value). (*P < 0.05)

**Fig. 4:**
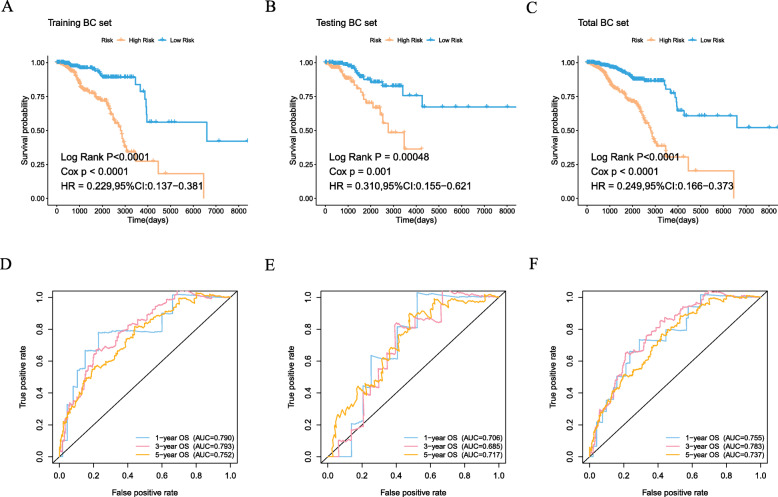
**a** Kaplan-Meier curve analysis of high-risk and low-risk patients in the training cohort. **b** Kaplan-Meier curve analysis of high-risk and low-risk patients in the testing cohort. **c** Kaplan-Meier curve analysis of high-risk and low-risk patients in the entire TCGA cohort. **d** Time-dependent ROC curve analysis of the training cohort. **e** Time-dependent ROC curve analysis of the testing cohort. **f** Time-dependent ROC curve analysis of the entire TCGA cohort

Among the identified 556 DEIGs, 66 prognostic DEIGs were identified by utilizing univariate cox regression analyses (Fig. 3a). KM survival analysis showed that 30 of them were significantly correlated with OS. TCGA BC samples were randomly separated into two sets (training set: validation set, 2:1). Then, lasso regression analysis was applied to increase the robustness and select the optimal variables based on training set. Finally, 15DEIGs were got for the construction of immune prognostic index based on the optimal value (Fig. 3b, c, Table 3). After the establishment of the immune prognostic model, BC patients were stratified into high-risk and low-risk subgroups based on the cut-off risk score (Fig. 3d, e). Heatmap was utilized to visualize the difference of gene expression profile in low- and high- risk patients in BC training set (Fig. 3f). The results from KM analysis revealed that high risk patients possessed lower overall survival in both training group and validation group (*P* < 0.001) (Fig. 4a, b, c). R software was utilized to draw the time-dependent ROC curvesand the AUC was calculated at different time points to estimate the predictive performance of our prognostic model in training cohort, testing cohort and the entire TCGA cohort. The ROC curve prompted that the risk scores model had dominant credibility and predictive value (AUC = 0.752, AUC = 0.704 for 5 years overall survival in training and validation group, respectively) (Fig. 4d, e, f).

**Table 3:** Multivariate cox regression analysis to establish RNA binding proteins risk prediction model

Gene	Coef
TSLP	−0.703829357640691
IL17B	−0.0870608394604504
NR3C2	−0.0255482484720901
RAC2	−0.130057137304801
SERPINA3	−0.0898937544948299
HSPA2	−0.120788735486787
CD79A	−0.0431127011058176
UNC93B1	0.513946621757904
NFKBIE	−0.329152003213528
SDC1	0.0854293362952585
IFNG	−0.220305753667004
IRF7	−0.171479153154717
GALP	2.91458293196349
TNFRSF18	−0.129391946165935
ULBP1	0.174787641983627

### Validation in external cohort and TCGA independent cohort

**Fig. 5:**
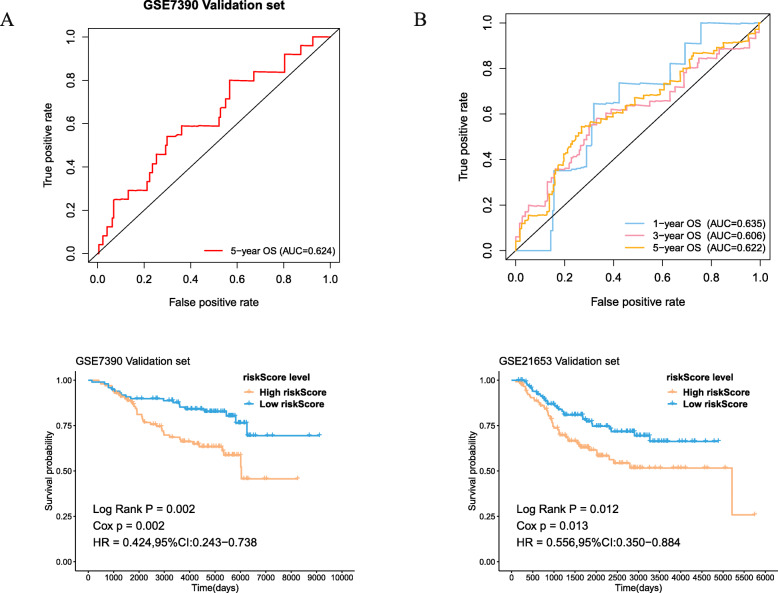
External validation set of the prognostic model. **a** ROC curve and AUC of the 15-gene signature in GSE7390 testing cohort. **b** ROC curve and AUC of the 15-gene signature in GSE21653 testing cohort. **c** KM survival analysis of the 15-gene signature in GSE7390 testing cohort. **d** KM survival analysis of the 15-gene signature in GSE21653 testing cohort

**Fig. 6:**
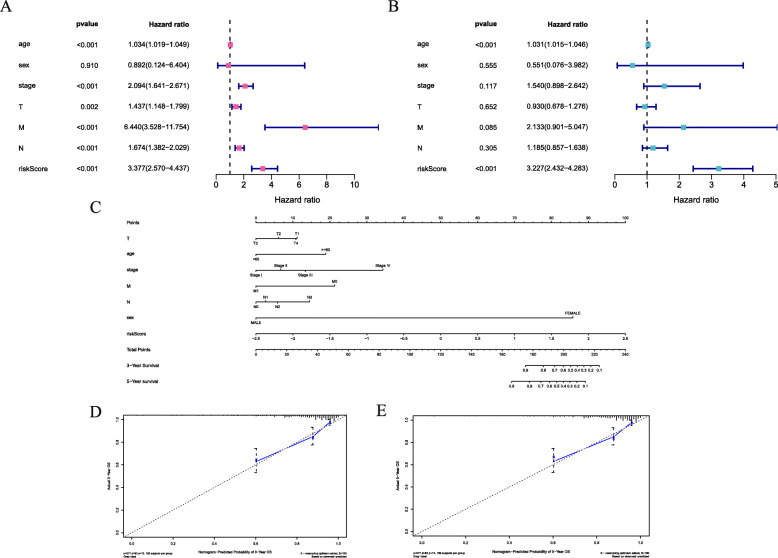
Cox’s proportional hazard model of correlative factors in breast cancer patients. **a** Univariate COX regression analysis for seven clinicopathological parameters affecting the overall survival. **b** Multivariate COX regression analysis for seven clinicopathological parameters affecting the overall survival. **c** An established nomogram to predict breast cancer survival based on cox model. **d**-**e** Plots displaying the calibration of each model comparing predicted and actual 3- and 5-year overall survival

To evaluate the operability and accuracy of the prognostic model in clinical practice, we further conducted the external validation analysis. As was shown in Fig. 5a and b, the AUC value was 0.624 for the 5-year OS in GSE7390 validation set and 0.635, 0.606, 0.622 for the 1-, 3-, 5-year OS, respectively, in GSE21653 validation set. What’s more, according to the KM curves, high risk scores were significantly associated with poor prognoses both in GSE7390 and GSE21653 validation set. (*P* = 0.002 and 0.012, respectively) (Fig. 5c, d) The results were consistent with those of the training set. In further univariate cox analysis, age, pathological stage, pathological T, N, M stage and high risk scores were associated with poor survival (Fig. 6a). In multivariate Cox model, only age and risk score worked as independent predicted factors (*P* < 0.001) (Fig. 6b). To establish a quantitative visualization model of breast cancer prognosis, multiple clinical factors were combined to establish a nomogram (Fig. 6c). The calibration of nomogram suggested that there was strong coherence between the predicted and actual 3- and 5-year overall survival (Fig. 6d, e).

### Recognition of gene sets for genome variation

Based on TCGA breast cancer data, we analyzed the copy number variation (CNV) of 15 model genes and showed the frequency of copy number variation through R-Circos package and R-ggplot2 package (Fig. S2A, Fig. S4). The results showed that the top three genes with the highest CNV frequency were RAC2, ULBP1, and SERPINA3. (Fig. S2B) Furthermore, we analyzed the single nucleotide polymorphism composition (SNPs) of 15 model genes (Fig. S2C). The results showed that NR3C2 had the most SNPs, including missense mutation and silent. Finally, the online tool website-cbioportal was utilized to analyze the genetic variation of 15 immune genes (Fig. S2D).

### Clinical and prognostic correlation of 15 model genes and the risk score

**Fig. 7:**
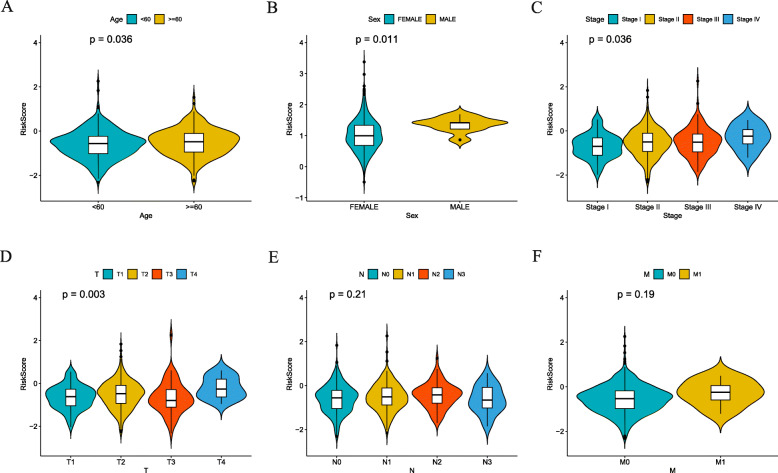
Correlation between immune genes risk scores and various clinical factors. **a** Age. **b** Sex. **c** Stage. **d** T stage. **e** N stage. **f** M stage

The proportion of 15 model genes in different clinical and pathological stages was investigated. Correlation analysis between tumor, node, metastasis stage, pathologic stage and 15 model genes expression in breast cancer cases were explored (Fig. S3A-D). Based on the results, it seemed that IL17B, NFKBIE and SERPINA3 mainly prompted the development of breast cancer. In addition, survival analysis showed that all model genes were significantly associated with survival (Fig. S4). Meanwhile, we found that the expression of RAC2, CD79A and IFNG were significantly associated with the infiltration of Macrophage M0 and Macrophage M2 (Fig. S5). Regard to the immune genes risk score, a strong correlation with age, sex, pathological stage and clinical T stage was identified (Fig. 7).

### Gene set enrichment analysis of risk scores

**Fig. 8:**
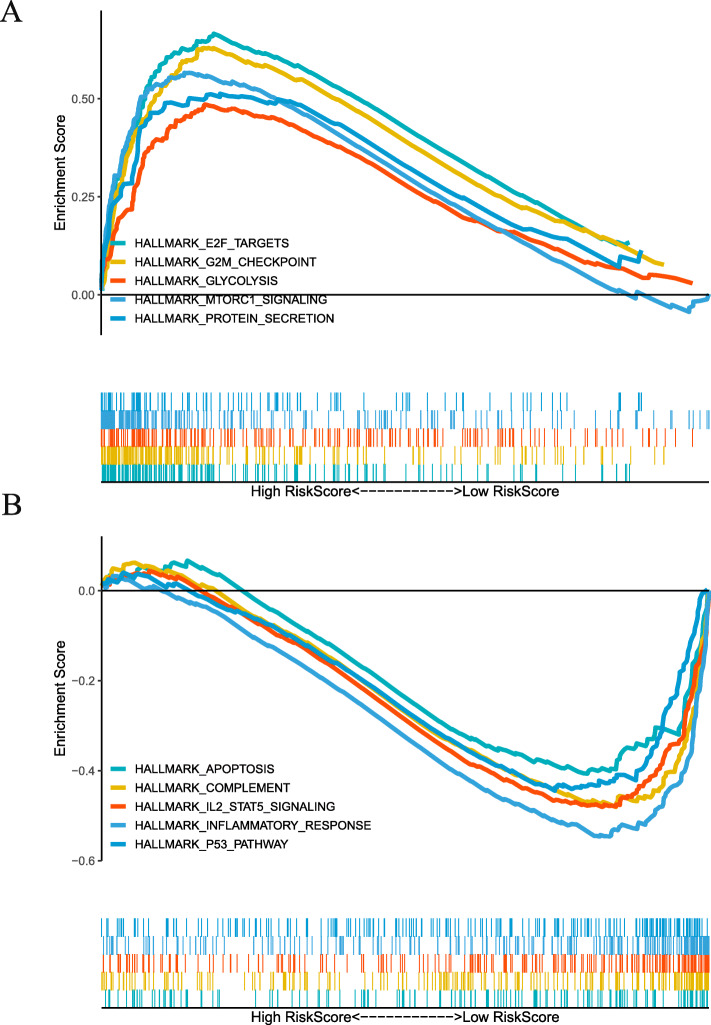
Gene set enrichment analysis of immune genes risk scores. **a** high risk scores. **b** low risk scores

To explore the biological correlation of risk scores involved in progression of breast cancer, a GSEA analysis of risk scores was performed based on the TCGA breast cancer cohort. GSEA analysis indicated high risk scores were associated with E2F_TARGETS, G2M_CHECKPOINT, GLYCOLYSIS, MTORC1_SIGNALING and PROTEIN_SECRETION pathway (Fig. 8a). In addition, low risk scores were associated with APOPTOSIS, COMPLEMENT, IL2_STAT5_SIGNALING, INFLAMMATORY_RESPONSE and P53 pathway (Fig. 8b).

## Discussion

BC is regarded as the most common malignant tumor in women. Although great efforts have been made to improve diagnosis and treatment strategies, it still poses a fatal threat to patients. Accumulation of evidence have shown that Cancer immunotherapy, especially the treatment of immune checkpoint inhibitors, has become an important part of the treatment of certain types of cancer, and has provided a continuous therapeutic effect for specific groups of patients [17]. Immune genes, such as cytokines, not only act locally, but rapidly spread within the tumor and affect the activation and dissemination of tumor immune cells [18, 19]. Obviously, different types of cancer have different immune gene subgroups. Therefore, the examination of immune gene subgroups is essential for judging the risk of tumors and exploring immunotherapy.

In our research, we performed a detailed and comprehensive evaluation of immune genes in BC. All gene expression data and patients clinical characteristics information were downloaded from TCGA dataset. Two thousand four hundred ninety-eight immune genes from ImmPort database were analyzed between breast cancer and normal tissues. Eventually, 556 DEIGs were verified. Moreover, we identified and constructed a 15 immune genes risk scores model for breast cancer through univariate and lasso regression analysis, including TSLP, IL17B, NR3C2, RAC2, SERPINA3, HSPA2, CD79A, UNC93B1, NFKBIE, SDC1, IFNG, IRF7, GALP, TNFRSF18 and ULBP1. Furthermore, to study the clinical and biological relevance of risk scores, the KM, ROC and GSEA analysis were conducted. Indeed, the high risk group received a lower survival, and possessed a higher histological grade.

Several DEIGs in the immune genes risk scores model have been investigated in human cancers. Thymic interstitial lymphopoietin (TSLP), a key inflammatory cytokine that induces type 2 inflammation, predicts a poor prognosis in oropharyngeal squamous cell carcinoma (OPSCC) [20]. With regard to breast cancer, Emma et al. has demonstrated that TSLP, which induced by IL-1 produced by breast tumors, act as a critical survival factor for the tumor [21]. This may indicate that TSLP can be a potential therapeutic intervention for breast cancer. Interleukin-17 (IL-17), a member of the interleukin family, is a cytokine that plays a role in inflammation and cancer, and can enhance lung cancer invasion/migration ability [22]. Seth et al. has found that the mammary tumor can induce IL17-producing γδ T cells, which can drive systemic expansion and polarization of neutrophils towards a CD8+ T cell-suppressive phenotype and subsequent metastasis formation in distant organs [23]. It seems that IL-17 plays a negative role in the prognosis of BC patients. In addition, studies have found that knocking down RAC2 can inhibit the progression of osteosarcoma by inhibiting the wnt signaling pathway [24]. Besides, the up regulation of hnRNP-K transcriptional activity mediated by SERPINA3 promotes the survival and proliferation of HCC cells, which may be an indicator of poor prognosis in HCC patients [25]. So far, overexpression of SERPINA3 has been observed in several cancer types including breast cancer and the high expression level has been demonstrated to positively correlate with poor prognosis in patients with breast cancer, which means SERPINA3 can be associated with a shorter OS [26]. AS a putative oncoprotein, Heat shock protein family a member 2 (HSPA2) is often up-regulated in human malignancies and promotes aggressive phenotype of tumors [27]. It seemed that overexpression of HSPA2 may be associated with worse clinical outcome. A recent study indicated that HSPA2 might play an important role in breast cancer development and progression by promoting cell growth, migration and invasion in xenografted mice [28]. However, it remains a controversy whether HSPA2 is a positive or negative regulator of carcinogenesis. NFKBIE aberrations are common genetic events in trans-b-cell malignancies, and NFKBIE deletion is a new marker of poor prognosis in primary mediastinal B-cell lymphoma (PMBL) [29]. The remaining genes have also been confirmed to be interrelated to malignant origin, aggressive behavior of tumors.

Similarly, Lai et al. [30] established a panel of 4 autophagy-related genes (ARG) signatures consisting of SERPINA1, ATG4A, NRG1 and IFNG to predict the prognosis of breast cancer, which can help clinicians make judgments and decisions on determining effective treatment strategies. Wang et al. [31] identified a six differentially-expressed genes (DEGs) model consisting of IGHA2, SERPINA1, GFALS, SPDYC, PAX7, and ADRB1 by using Cox regression survival modeling for breast cancer. In another study [32], the authors constructed a prognostic risk scoring system containing 6 genes (SCUBE3, RDH16, SPC24, SPC25, CCDC69 and DGAT2), suggesting that these mRNAs may serve a driving role in the progression of Her2-positive BC. The construction of this risk scoring system is conducive to identifying high-risk HER2-positive BC patients, and it subserve to help achieve personalized targeted therapy. Different from previous studies, our study provides novel insights into the role of Immune-related genes in the genesis and progression of BC. We first focused on DEIGs, and established and verified a novel DEIGs risk scores prediction model. And our prognostic model showed good predictive performance with regard to survival, which may contribute to the development of new prognostic indicators for BC. Besides, the Immune-related gene marker showed strongly association with immunoinfiltrating cells, which demonstrates that these Immune-related genes could be used in clinical adjuvant treatments.

Nevertheless, there still remain some weak points in our research. Firstly, our results are based on bulk RNA sequencing of single omics. The heterogeneity and diversity between cells in the tumor microenvironment is ignored. Secondly, only gene expression and gene mutation levels are concerned, while tumor burden, methylation levels and other equally important events in tumor progression are ignored.

## Conclusion

In conclusion, our study reveals the biological effects of immune genes in the origin and development of BC. The immune gene risk score model has advantages in predicting the prognosis of BC, which is an independent factor affecting the prognosis of BC. In addition, our findings may be of great guiding value in make a thorough inquiry of novel strategies for cancer immunological diagnosis and treatment. With the rapid development of high-throughput sequencing technology, it is reasonable to believe that this scoring system can provide recommendations for patients’ immune status as well as clinical risk assessment and treatment strategies.

## Supplementary Information

**Additional file 1: Figure S1.** GO (A) and KEGG(B) enrichment analysis of DEIGs.

**Additional file 2: Figure S2.** Analysis of copy number variation and single nucleotide polymorphism of 15 model immune genes.

**Additional file 3: Figure S3.** Correlation analysis between TNM&Stage and 15 model genes in breast cancer cases. (A) Correlation analysis between tumor stage and 15 model genes expression in breast cancer cases. (B) Correlation analysis between node stage and 15 model genes expression in breast cancer cases. (C) Correlation analysis between metastasis stage and 15 model genes in breast cancer cases. (D) Correlation analysis between pathologic stage and 15 model genes expression in breast cancer cases.

**Additional file 4: Figure S4.** Survival analysis of 15 model immune genes.

**Additional file 5: Figure S5.** Correlation between 15 model immune genes and immune cell infiltration.

## Data Availability

The data was available in the ImmPort database (https://www.immport.org/) and The Cancer Genome Atlas (TCGA) database (https://cancergenome.nih.gov/).

## Abbreviations

OS: Over survival
TNBC: Triple negative breast cancer
ER: Estrogen receptor
PR: Progesterone receptor
HER-2: Human epidermal growth factor receptor 2
TIICs: Tumor infiltrating immune cells
KEGG: Kyoto Encyclopedia of Genes and Genomes
GO: Gene ontology
DAVID: Database for Annotation, Visualization, and Integrated Discovery
GSEA: Gene enrichment analysis
CNV: Copy number variation
LASSO: Least absolute shrinkage and selection operator
DEIGs: Differentially expressed immune genes
SNPs: Single nucleotide polymorphism
TME: Tumor micro-environment
TSLP: Thymic stromal lymphopoietin
OPSCC: Oropharyngeal squamous cell carcinoma
HSPA2: Heat shock protein family a member 2
PMBL: Primary mediastinal B-cell lymphoma
ARGs: Autophagy-related genes
DEGs: Differentially expressed genes
