# Supplementary figures and images for: Identification and development of an independent immune-related genes prognostic model for breast cancer
Source: BMC Cancer. 2021 Mar 30;21:329. doi: 10.1186/s12885-021-08041-x (PMC8011146; doi:10.1186/s12885-021-08041-x)

A

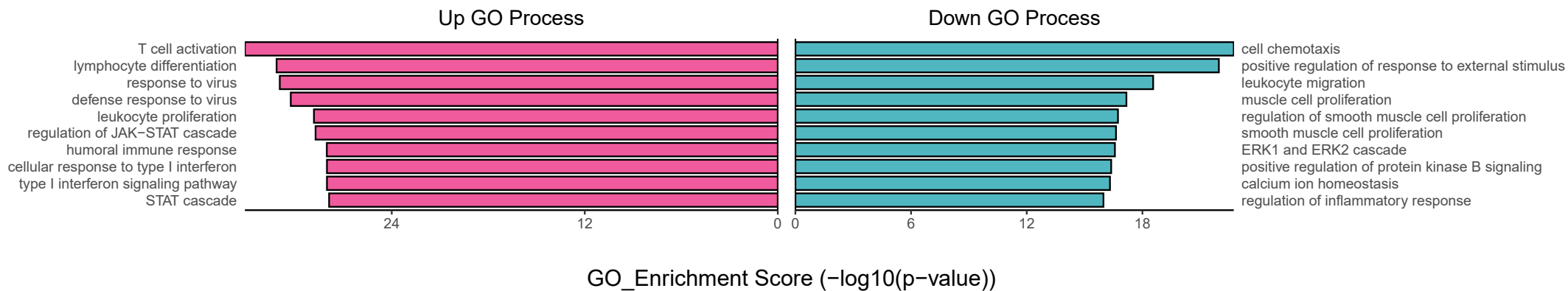

B

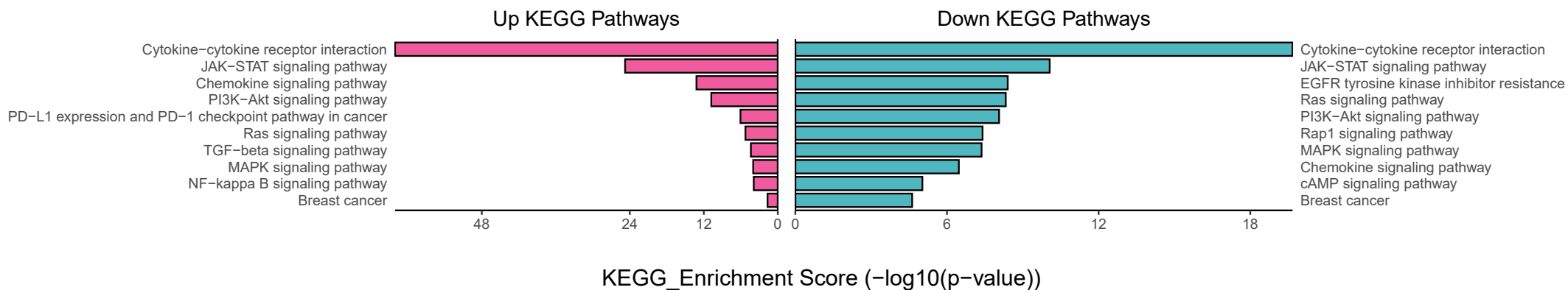

Supplement: Supplementary file 1 — Additional file 1: Figure S1. GO (A) and KEGG(B) enrichment analysis of DEIGs. [file 12885_2021_8041_MOESM1_ESM.pdf]

A

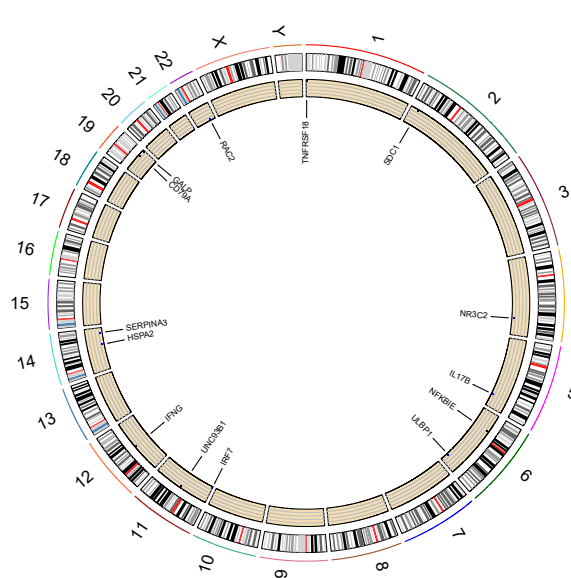

B

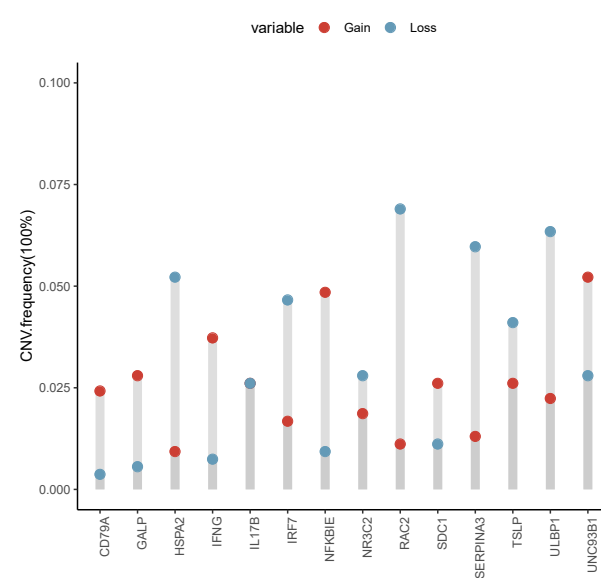

C

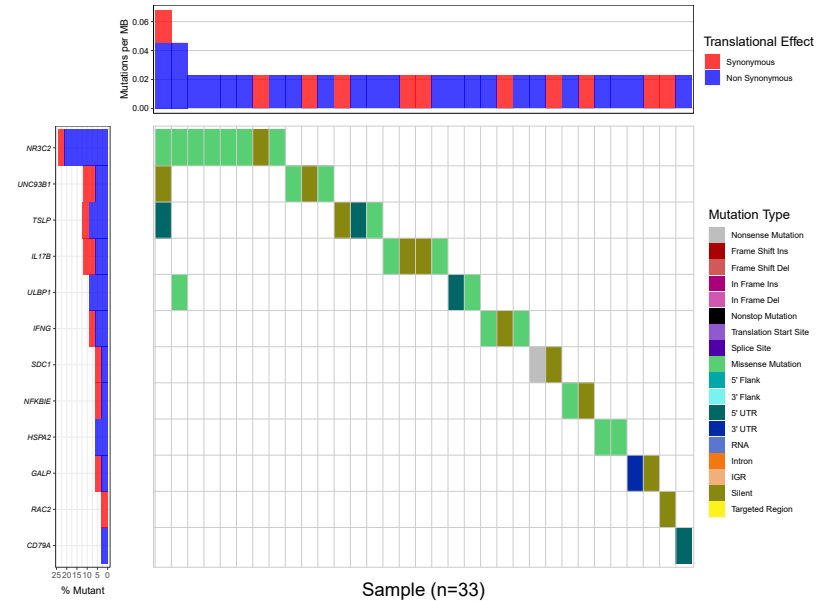

D

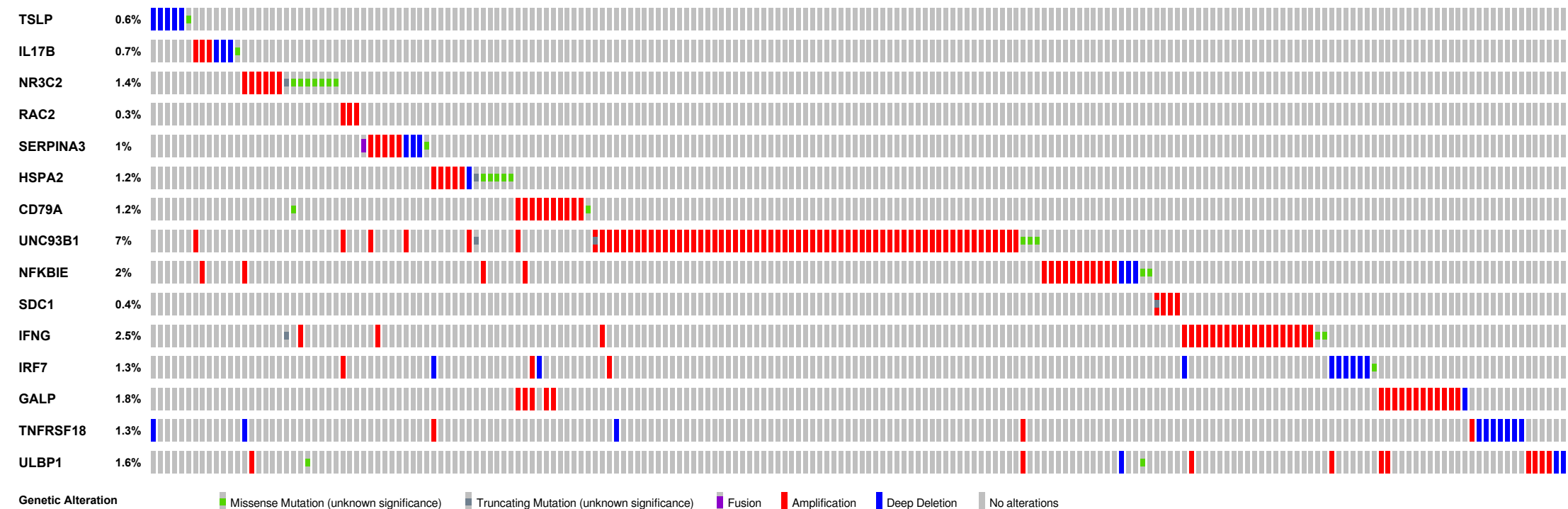

Supplement: Supplementary file 2 — Additional file 2: Figure S2. Analysis of copy number variation and single nucleotide polymorphism of 15 model immune genes. [file 12885_2021_8041_MOESM2_ESM.pdf]

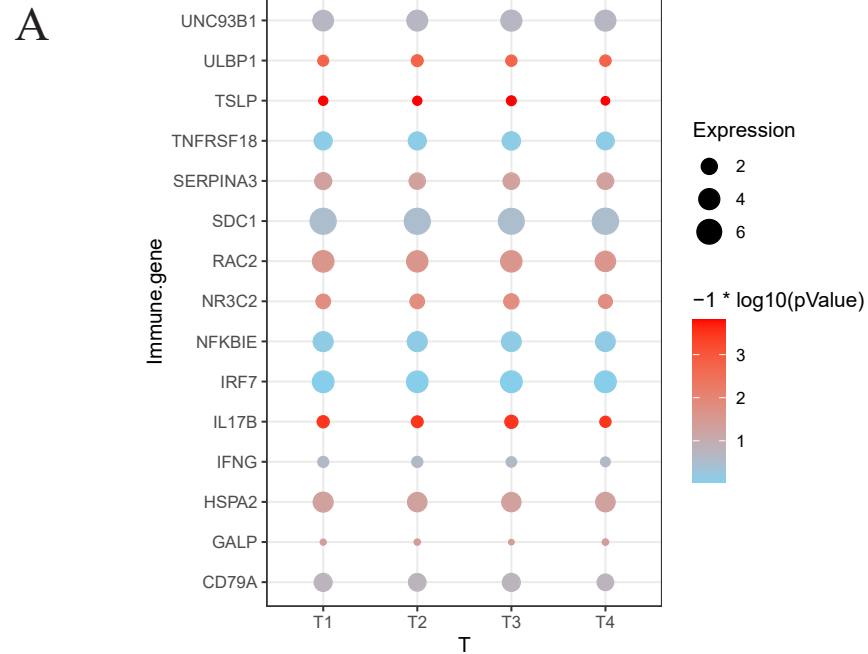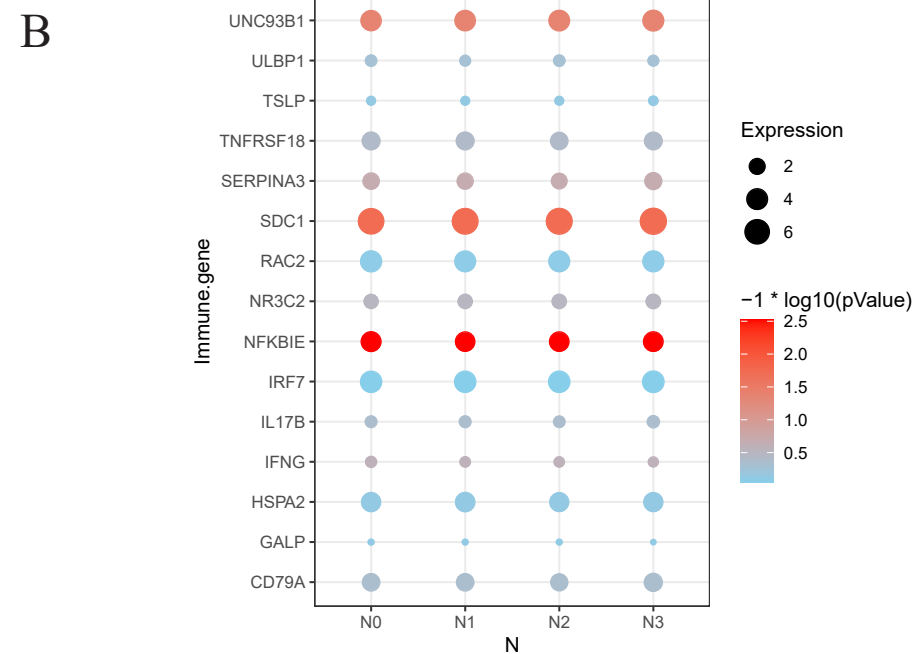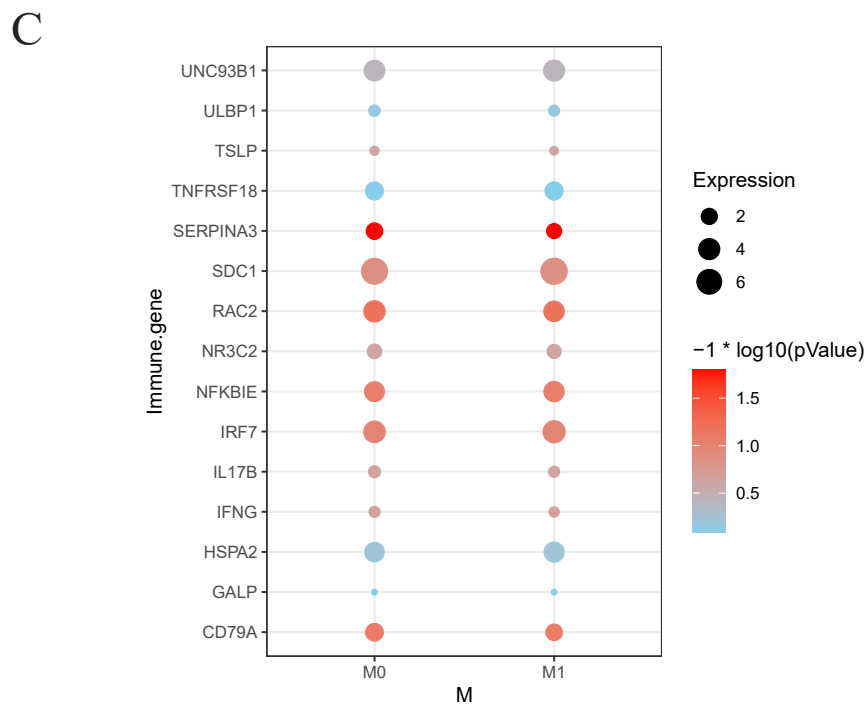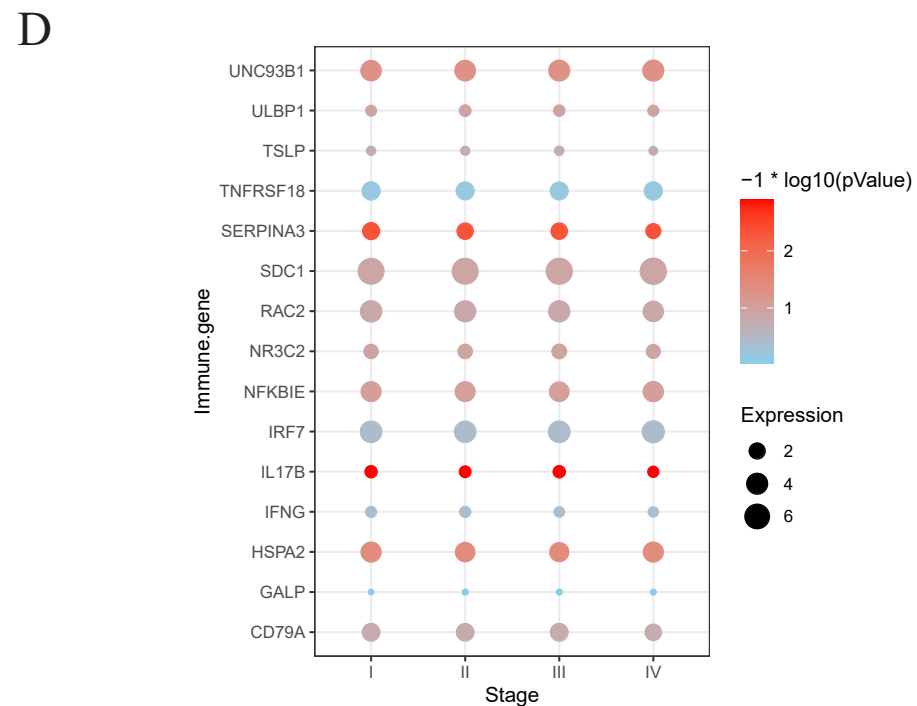

Supplement: Supplementary file 3 — Additional file 3: Figure S3. Correlation analysis between TNM&Stage and 15 model genes in breast cancer cases. (A) Correlation analysis between tumor stage and 15 model genes expression in breast cancer cases. (B) Correlation analysis between node stage and 15 model genes expression in breast cancer cases. (C) Correlation analysis between metastasis stage and 15 model genes in breast cancer cases. (D) Correlation analysis between pathologic stage and 15 model genes expression in breast cancer cases. [file 12885_2021_8041_MOESM3_ESM.pdf]

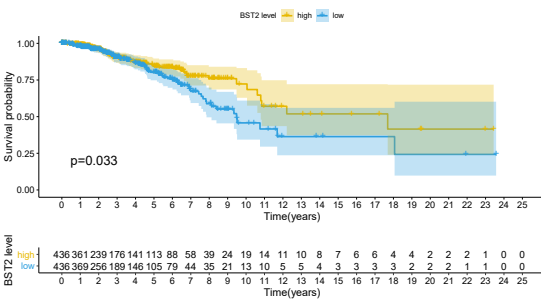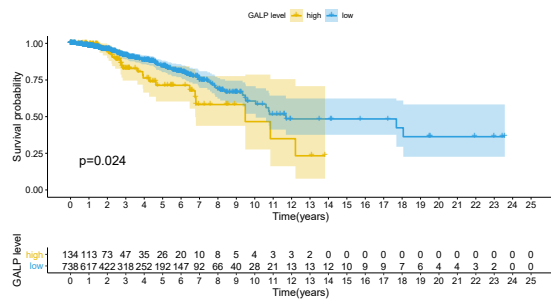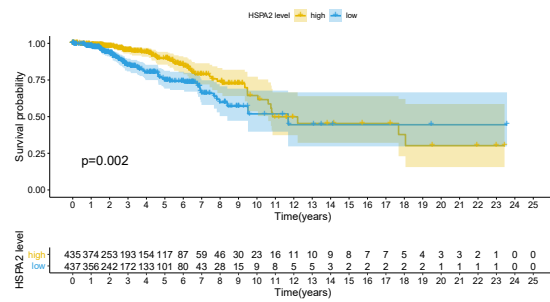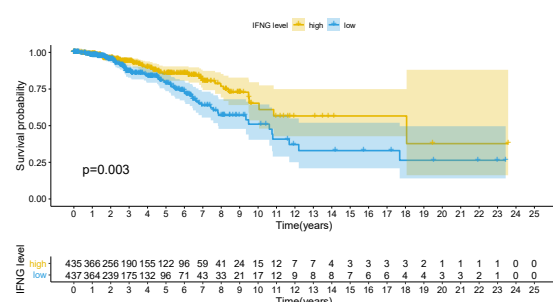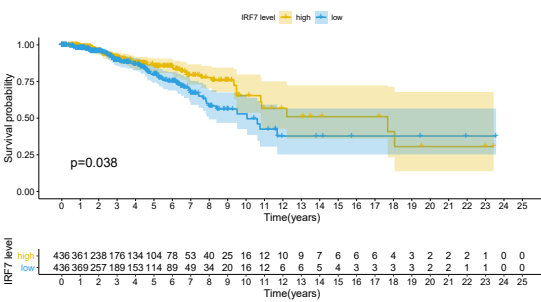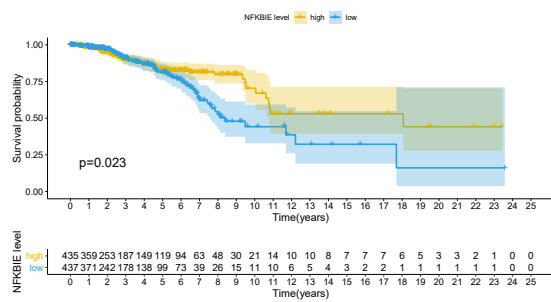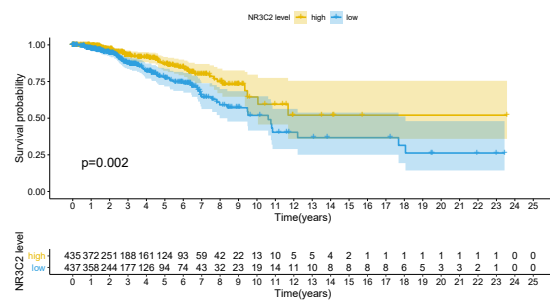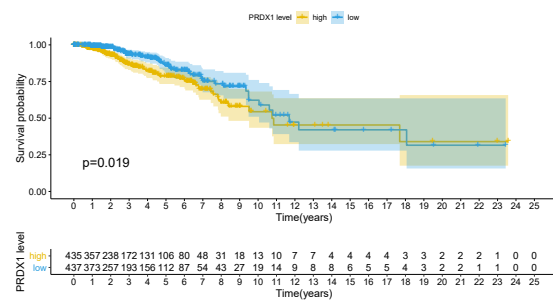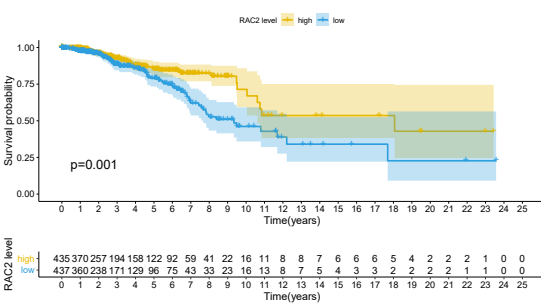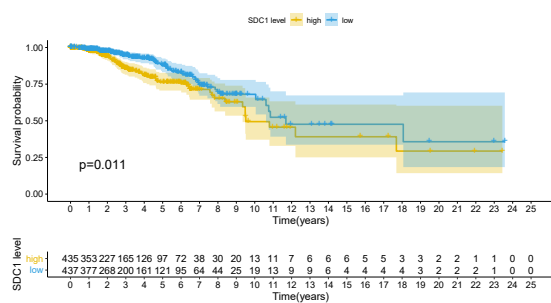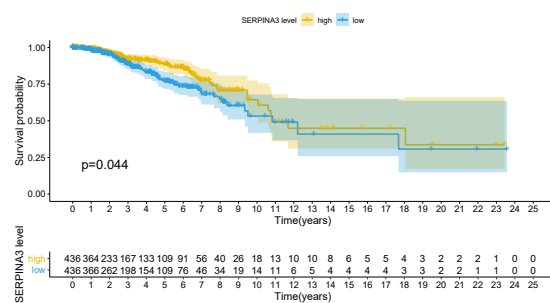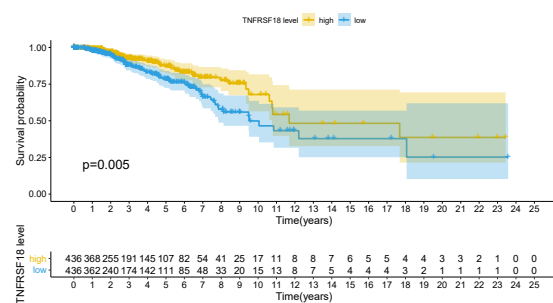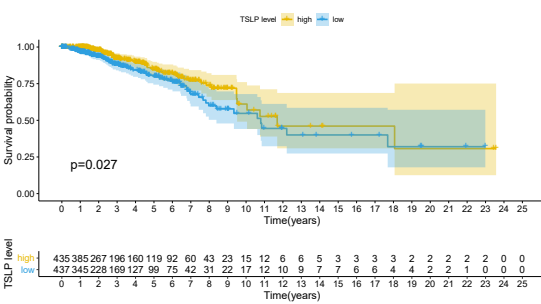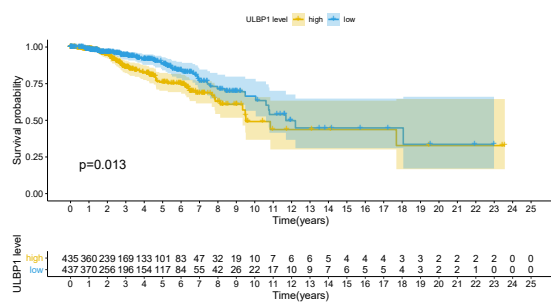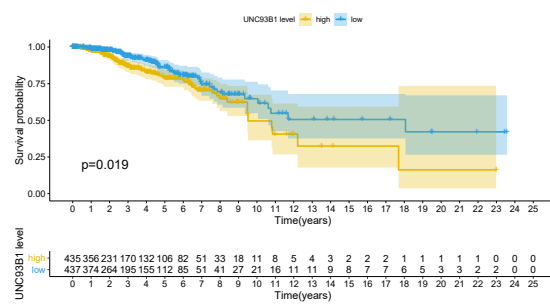

Supplement: Supplementary file 4 — Additional file 4: Figure S4. Survival analysis of 15 model immune genes. [file 12885_2021_8041_MOESM4_ESM.pdf]

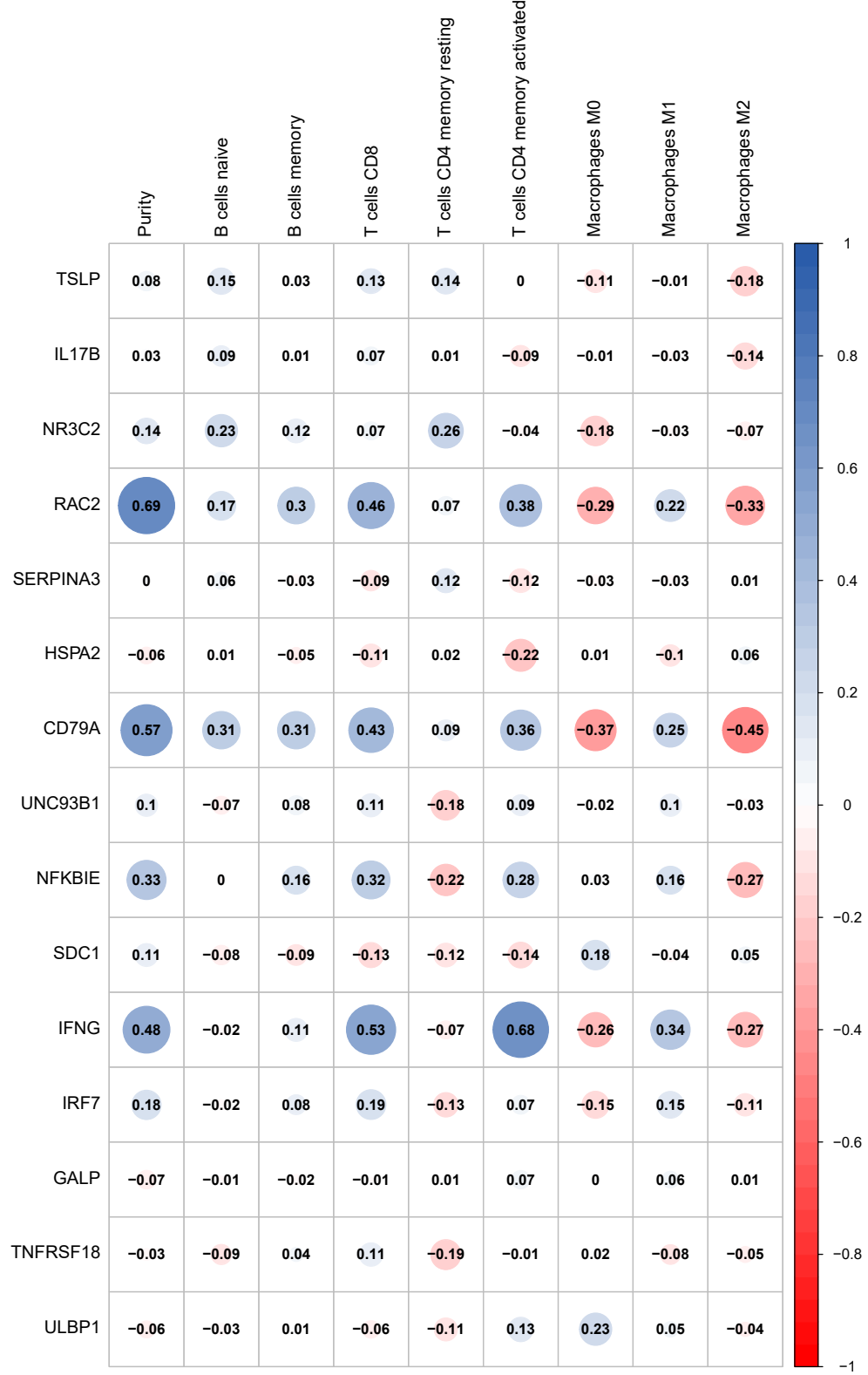

Supplement: Supplementary file 5 — Additional file 5: Figure S5. Correlation between 15 model immune genes and immune cell infiltration. [file 12885_2021_8041_MOESM5_ESM.pdf]
